# Genotype–Phenotype Correlations and Their Implications for Personalized Genetic Counseling in 103 Patients With *PRRT2* Mutations

**DOI:** 10.1155/humu/8968480

**Published:** 2026-10-05

**Authors:** Shangyu Wang, Xiaohui Min, Gege Yuan, Mingying He, Bing Wu, Xiaoyu Liu, Bixia Zheng, Yang Li, Aihua Zhang, Gang Zhang

**Affiliations:** ^1^ Medical School Nanjing University Nanjing China nju.edu.cn; ^2^ Department of Neurology Children′s Hospital of Nanjing Medical University Nanjing China; ^3^ Nanjing Key Laboratory of Pediatrics Children′s Hospital of Nanjing Medical University Nanjing China

**Keywords:** genotype–phenotype, infantile convulsions with paroxysmal choreoathetosis, paroxysmal kinesigenic dyskinesia, *PRRT2*, self-limited familial infantile epilepsy

## Abstract

**Objective:**

*PRRT2* mutations are associated with a wide spectrum of paroxysmal neurologic disorders. In this study, we analyze clinical and genetic data from a large pediatric cohort to define genotype–phenotype correlations and to provide a basis for individualized genetic counseling.

**Methods:**

This retrospective study included 103 children with *PRRT2* mutations treated at the Children′s Hospital Affiliated to Nanjing Medical University from August 2018 to August 2023. Detailed clinical data and genetic results were collected and systematically analyzed.

**Results:**

Among the 103 children, self‐limited familial infantile epilepsy (SeLFIE) was the most common phenotype. A total of 26 different *PRRT2* mutations were identified, 93.2% of which were predicted loss‐of‐function (LOF) mutations. These mutations were highly enriched in the N‐terminal cytoplasmic region of the protein, particularly in the proline‐rich domain. Predicted LOF mutations accounted for nearly all cases of SeLFIE and infantile convulsions with paroxysmal choreoathetosis. Clinical features, including age of onset, rates of abnormal EEG and brain MRI findings, and treatment response, showed a gradient across the three phenotypic groups, with the most pronounced abnormalities in SeLFIE and the mildest in paroxysmal kinesigenic dyskinesia. Additionally, 12 novel variants were identified.

**Conclusion:**

This study confirms that *PRRT2*‐associated disorders are primarily driven by predicted LOF mutations and, using a large cohort, establishes a genotype‐phenotype‐treatment response continuum model based on residual PRRT2 protein activity. This model provides a critical framework for prognosis assessment and personalized genetic counseling.

## 1. Introduction

*PRRT2* (OMIM: 614386), located on chromosome 16p11.2, contains four exons with exons 2–4 encoding a 340‐amino‐acid multidomain protein. It was identified in 2011 as the causative gene for paroxysmal kinesigenic dyskinesia (PKD) [1]. The spectrum of paroxysmal disorders associated with *PRRT2* mutations is broad, with self‐limited familial infantile epilepsy (SeLFIE) considered the most common phenotype in early life [2]. Infantile convulsions with paroxysmal choreoathetosis (ICCA) manifests as paroxysmal dyskinesia appearing in childhood or adolescence following the remission of infantile seizures [3]. In addition to the core phenotypes of epilepsy and paroxysmal dyskinesia, other rare presentations including migraine/headache, episodic ataxia, and isolated intellectual disability have also been reported. Although seizures generally show spontaneous remission with a favorable prognosis, other symptoms such as dyskinesias often require medical management [4].

Most reported *PRRT2* mutations are truncating variants that result in haploinsufficiency [5]. Despite the growing list of identified *PRRT2* alterations, the full clinical and genetic spectrum of the associated disorders remains incompletely characterized. Prior studies have largely focused on individual phenotypes, with few systematic, crosscohort analyses covering the entire disease continuum. Thus, a comprehensive evaluation of the clinical and genetic features across the full spectrum of *PRRT2*‐related conditions is urgently needed.

## 2. Materials and Methods

### 2.1. Participant Recruitment and Inclusion/Exclusion Criteria

This study enrolled children with confirmed *PRRT2* mutations who were diagnosed at the Children′s Hospital of Nanjing Medical University between August 2018 and August 2023. All participants underwent genetic testing, and data were systematically collected and analyzed.

Inclusion criteria were as follows: (i) fulfillment of clinical diagnostic criteria for *PRRT2*‐related disorders—including epilepsy or PKD—according to the 2017 International League Against Epilepsy (ILAE) classification and previous literature [6]; (ii) completion of or consent to genetic testing, with results confirming the presence of *PRRT2* mutations as the sole genetic variant; and (iii) age under 18 years.

Exclusion criteria comprised (i) poor family compliance, lack of cooperation, or incomplete clinical data and (ii) confirmed acquired brain disorders with corresponding structural abnormalities, or etiologies of immune, infectious, or organic origin.

### 2.2. Clinical Data Collection

For each enrolled child with *PRRT2* mutations, we extracted data on the following variables: sex, age at disease onset, family history, developmental milestones before and after onset, seizure semiology and frequency before and after treatment, specific neurological signs, comorbid conditions, remission course, and results of electroencephalography (EEG), neuroimaging, and genetic testing performed at our institution. Seizure types were classified according to the 2017 ILAE epilepsy classification criteria [6], based on a synthesis of medical records, parental reports, and available video recordings. All antiseizure medications and treatment regimens used during the diagnostic and therapeutic course were systematically documented. Disease progression and remission status were evaluated from treatment initiation through the most recent follow‐up, with a minimum observation period of 2 years, to assess both short‐term efficacy and long‐term prognosis.

Treatment response was defined as either complete seizure freedom for at least 6 months following the initiation of therapy or at the most recent follow‐up or a reduction in baseline seizure frequency exceeding 50%. Treatment was deemed ineffective if the reduction in seizure frequency was less than 50% or if no clinical response was observed. For patients with the PKD phenotype, response to antiseizure medications was further stratified into three levels: (i) complete response—total abolition of all attacks, with or without persistent aura; (ii) partial response—persistence of only rare, low‐frequency attacks that did not interfere with daily functioning; and (iii) nonresponse—a reduction in attack frequency of less than 25% relative to the pretreatment year.

According to the 2010 ILAE definition of drug‐resistant epilepsy, treatment failure—and thus drug resistance—is established when a patient continues to experience seizures of any semiology, regardless of precipitating factors, for a duration of at least 1 year, despite having received two appropriately selected antiseizure medications (ASMs). These medications must have been administered either sequentially as monotherapy or in combination, at adequate dosages and for sufficient durations, with documented tolerability and, where applicable, therapeutic drug levels within the effective range [7].

The researchers assessed the developmental level based on parental descriptions and the child′s clinical developmental status. An initial evaluation was conducted using the child development schedule and developmental milestone guidelines from the “Child Health” chapter of the ninth edition of *Pediatrics*. Detailed descriptions from parents regarding the child′s developmental level after disease onset were carefully recorded. Additionally, the researchers calculated the developmental quotient (developmental age/chronological age ^∗^100) based on the actual language abilities and gross motor developmental milestones of children with *PRRT2*‐related disorders. The severity of developmental delay was broadly categorized as profound (DQ < 25), severe (25 ≤ DQ ≤ 39), moderate (40 ≤ DQ ≤ 54), or mild (55 ≤ DQ ≤ 75).

### 2.3. Genetic Analysis

Exome sequencing was performed using the IDT xGen Exome Research Panel v2.0. Detected variants were aligned against the human reference genome (GRCh37/hg19) via the UCSC Genome Browser and annotated by crossreferencing public databases, including dbSNP, the Genome Aggregation Database (gnomAD), the 1000 Genomes Project, and the Human Gene Mutation Database (HGMD). Variant pathogenicity was further assessed using multiple in silico prediction tools: Missense variants were evaluated using SIFT, PolyPhen‐2, and MutationTaster; splicing and intronic variants were assessed using SpliceAI and dbscSNV; CADD scores were also used as supporting evidence.

All enrolled patients presented with clinical phenotypes consistent with *PRRT2*‐related disorders and were confirmed to carry *PRRT2* mutations by genetic testing. We retrospectively reviewed their WES data and specifically interrogated variants in *PRRT2* (NM_145239.3). Candidate variants were prioritized according to the following criteria: (1) located in *PRRT2*; (2) minor allele frequency < 0.01 in public population databases (gnomAD, 1000 Genomes); (3) classified as pathogenic (P), likely pathogenic (LP), or variant of uncertain significance (VUS) according to the ACMG/AMP guidelines; and (4) confirmed by Sanger sequencing with available family members for cosegregation analysis. Following the ACMG/AMP guidelines, variants were classified into five categories: P, LP, VUS, likely benign (LB), and benign (B).

All candidate variants were validated via Sanger sequencing on an ABI 3730 capillary sequencer following PCR amplification. Primer sequences and PCR conditions are available from the corresponding author upon reasonable request. Evolutionary conservation analysis of the PRRT2 protein was performed by aligning homologous sequences from eight representative species—including *Mus musculus*, *Rattus norvegicus*, *Pan troglodytes*, *Macaca mulatta*, *Canis lupus familiaris*, *Mustela putorius furo*, *Capra hircus*, and *Myotis brandtii*—downloaded from the NCBI database, with the human reference protein sequence (NP_660282.2). Hydrogen bond interaction analysis was performed using PyMOL software to assess the structural impact of missense mutations based on the wild‐type human PRRT2 protein sequence (UniProt: Q7Z6L0.1.A).

To predict the functional impact of the identified variants, we referred to the ACMG/AMP guidelines. Frameshift variants, canonical splice‐site variants affecting the invariant ± 1 or 2 dinucleotides, *PRRT2* Exon 2 deletions, and whole‐gene deletions were classified as predicted loss‐of‐function (LOF) based on the PVS1 criterion, as haploinsufficiency is the established pathogenic mechanism for *PRRT2*‐related disorders, and no evidence of NMD escape or retained C‐terminal function was identified for these variants. Noncanonical splice‐site variants (e.g., deep intronic variants), missense, and synonymous variants were classified as predicted non‐LOF, as they could not be definitively assigned as LOF based solely on variant type. For variants in the non‐LOF group, we further applied ACMG evidence codes—including PM2 (population frequency), PP3 (in silico prediction), BS3 (functional studies showing no damaging effect), and PS3 (functional studies confirming a damaging effect)—with functional evidence derived from published studies [8, 9].

### 2.4. Statistical Methods

Statistical analysis was performed using SPSS 25.0 software. Normality of continuous variables was assessed using the Shapiro–Wilk test. As the data were not normally distributed (*p* < 0.05), continuous variables were expressed as median (interquartile range, IQR) and compared using the Mann–Whitney *U* test (for two‐group comparisons) or the Kruskal–Wallis test (for multigroup comparisons). Categorical variables were expressed as frequency and percentage (*n*, %) and compared using the Chi‐square test or Fisher′s exact test when expected cell counts were < 10. A *p* value < 0.05 was considered statistically significant.

## 3. Results

### 3.1. Clinical Features

This study summarized the clinical data of 103 pediatric patients with *PRRT2* mutations, including 62 males and 41 females. Among them, 20 cases were sporadic and 83 were familial. SeLFIE (70/103, 68.0%) was the most common phenotype, followed by PKD (22/103, 21.4%) and ICCA (11/103, 10.7%). Males showed a significant predominance in the PKD group (86.4%, *p* < 0.01). In the SeLFIE cohort, the median age of seizure onset was 4.0 months (IQR: 4.0–6.0 months), with a range of 3 months to 4 years and 7 months. In the ICCA cohort, the median age of epilepsy onset was 9.0 months (IQR: 6.0–12.0 months), ranging from 4 months to 2 years, whereas the median age of dyskinesia onset was 7.0 years (IQR: 5.0–9.0 years), ranging from 4 to 12 years. No significant difference was observed in the age of epilepsy onset between the SeLFIE and ICCA groups (*p* = 0.18).

In the PKD cohort, the median age of onset was 10.0 years (IQR: 8.0–12.0 years), with a range of 4 years and 3 months to 13 years and 7 months. The age of dyskinesia onset in the PKD group was significantly later than that in the ICCA group (*p* = 0.016). Among patients with PKD, 14 reported prodromal symptoms, most commonly abnormal sensations in the initially affected limb prior to attack onset, with sudden movement identified as the predominant trigger. Dystonia was observed in nine cases, chorea in nine cases, and mixed‐type attacks in four cases. In this cohort, approximately 13.6% of patients whose initial manifestation was epilepsy subsequently developed dyskinesia during childhood, with a median interval of approximately 7 years between the two conditions. Conversely, roughly 33.3% of patients presenting with childhood‐onset dyskinesia had a documented history of epilepsy in early life.

Regarding auxiliary examinations, abnormal EEG findings were most prevalent in the SeLFIE group (61.4%), whereas no EEG abnormalities were detected in the PKD group. Abnormal brain MRI findings were significantly more frequent in the SeLFIE (58.6%) and ICCA (54.5%) groups than in the PKD group (13.6%) (all *p* < 0.001). This observation suggests that even benign epilepsy syndromes may be associated with substantial structural or functional cerebral changes, underscoring the need for long‐term neurological surveillance. Analysis of treatment outcomes demonstrated that the seizure control rate was significantly higher in the SeLFIE group compared with the ICCA group (94.3% vs. 72.7%) (Table 1).

**Table 1:** Demographic and baseline clinical characteristics of children with different *PRRT2*‐related phenotypes.

Demographic characteristics	Population (*N* = 103)	SeLFIE (*n* = 70)	ICCA (*n* = 11)	PKD (*n* = 22)	*p*
Sex (%)					<0.01
Female	41 (39.8%)	34 (48.6%)	4 (36.4%)	3 (13.6%)	
Male	62 (60.2%)	36 (51.4%)	7 (63.6%)	19 (86.4%)	
Age of seizure onset (months)	N/A	4.0 (4.0, 6.0)	9.0 (6.0, 12.0)	—	0.18
Onset age of dyskinesia (years)	N/A	N/A	7.0 (5.0, 9.0)	10.0 (8.0, 12.0)	0.016
Positive family history (%)	33 (32.0%)	25 (35.7%)	5 (45.5%)	3 (13.6%)	0.085
Intellectual disability (%)	12 (11.7%)	9 (12.9%)	2 (18.2%)	1 (4.5%)	0.387
Motor delay (%)	4 (3.9%)	3 (4.3%)	1 (9.1%)	0 (0.0%)	0.374
Language delay (%)	12 (11.7%)	10 (14.3%)	2 (18.2%)	0 (0.0%)	0.108
Behavioral problems (ASD, ADHD, etc, %)	11 (10.7%)	8 (11.4%)	2 (18.2%)	1 (4.5%)	0.609
First EEG (abnormal, %)	48 (46.6%)	43 (61.4%)	5 (45.5%)	0 (0.0%)	< 0.001
Brain MRI (abnormal, %)	50 (48.5%)	41 (58.6%)	6 (54.5%)	3 (13.6%)	< 0.001
Seizure therapeutic response (%)					0.148
Seizure free	N/A	66 (94.3%)	8 (72.7%)	N/A	
Seizure reduction	N/A	1 (1.4%)	1 (9.1%)	N/A	
Seizure poor control	N/A	3 (4.3%)	2 (18.2%)	N/A	
Dyskinesia therapeutic response (%)					0.647
Dyskinesia free	N/A	N/A	5 (45.5%)	9 (40.9%)	
Dyskinesia reduction	N/A	N/A	6 (54.5%)	12 (54.6%)	
Dyskinesia poor control	N/A	N/A	0 (0.0%)	1 (4.5%)	
Hotspot mutation (c.649_650insC, %)	48 (46.6%)	28 (40.0%)	7 (63.6%)	13 (59.1%)	0.180
Severity of functional impairment					0.820
Predicted LOF mutations	96 (93.2%)	66 (94.3%)	10 (90.9%)	20 (90.9%)	
Predicted non‐LOF mutations	7(6.8%)	4 (5.7%)	1 (9.1%)	2 (9.1%)	
Inheritance					0.064
De novo	20 (19.4%)	18 (25.7%)	1 (9.1%)	1 (4.5%)	
Familial	83 (80.6%)	52 (74.3%)	10 (90.9%)	21 (95.5%)	

*Note:* Data are presented as *n* (%) for categorical variables and as median (interquartile range, IQR) for continuous variables. Categorical variables were compared using the chi‐square test or Fisher′s exact test when expected cell counts were < 10. Continuous variables were compared using the Mann–Whitney *U* test or the Kruskal–Wallis test. *p* < 0.05 was considered statistically significant. Normality was assessed using the Shapiro–Wilk test; nonparametric tests were applied because of nonnormal distribution of the data.

### 3.2. *PRRT2* Screening

Among the 103 patients, 26 distinct *PRRT2* mutations were identified (Figure 1). The predicted LOF variants accounted for the majority of cases, being present in 96 patients (93.2%). These predicted LOF variants included four subtypes: frameshift mutations, which were the most frequent, occurring in 76 cases (73.8%) and representing 17 distinct types; CNVs involving exon 2 deletions of *PRRT2*, observed in 16 cases (15.5%); whole‐gene deletions, detected in three cases (2.9%); and one canonical splice‐site variant (c.‐65‐2A>G) in one case (1.0%). The remaining seven patients (6.8%) harbored predicted non‐LOF variants, which included missense mutations in five cases, splice‐site mutations in one case, and a single synonymous mutation in one case (Figure 2D).

Among the identified variants, two affected splice sites (c.‐65‐2A>G and c.880‐34G>A), five were missense changes (c.236C>T/p.Ser79Leu, c.640G>C/p.Ala214Pro, c.826T>A/p.Cys276Ser, c.865G>C/p.Ala289Pro, and c.916G>A/p.Ala306Thr), and one was synonymous (c.1011C>T/p.Gly337Gly). The frameshift insertion c.649_650insC (p.Arg217Profs ^∗^8) emerged as the most frequent pathogenic allele, accounting for nearly half of all cases. This variant was observed across all clinical phenotypes examined, with no significant intergroup differences in its distribution. Importantly, we identified 12 novel variants not previously reported in the literature (Table 2), thereby substantially expanding the known mutational spectrum of *PRRT2*.

**Table 2:** *PRRT2* mutations and genotype–phenotype correlations in the study cohort.

Number of cases (%)	Nucleotide change	Amino acid change	Type of mutation	Exon/IVS	Inheritance	Phenotype (*n*)	Previously reported/novel	ACMG	ACMG criteria	Polyphen‐2	SIFT	MutationTaster
48 (46.6%)	c.649_650insC	p.Arg217Profs ^∗^8	Frameshift	Exon2	De novo/familial	SeLFIE (28)/ICCA (7)/PKD (13)	Known as SeLFIE, ICCA, and PKD (PMID:22120146)	P	PVS1 + PP1	—	—	DC
16 (15.5%)	*PRRT2* exon2 deletion	—	CNV	—	De novo/familial	SeLFIE (15)/ICCA (1)	Known as SeLFIE, ICCA, and PKD (PMID:32237035)	P	PVS1 + PS2 + PM2	—	—	DC
6 (5.8%)	c.649delC	p.Arg217Glufs ^∗^12	Frameshift	Exon2	Familial	SeLFIE (4)/ICCA (1)/PKD (1)	Known as SeLFIE, ICCA, and PKD (PMID:24755245, 24370076)	P	PVS1 + PP1 + PS2	—	—	DC
6 (5.8%)	c.641dupC	p.Arg217Profs ^∗^8	Frameshift	Exon2	De novo/familial	SeLFIE (5)/PKD (1)	Known as PKD (PMID:22101681)	P	PVS1 + BS1	—	—	DC
4 (3.9%)	c.640_641insC	p.Ala214Alafs ^∗^11	Frameshift	Exon2	Familial	SeLFIE	Known as SeLFIE, ICCA, and PKD (PMID:22120146,37228410)	P	PVS1 + PP1	—	—	DC
3 (2.9%)	*PRRT2* gene deletion	—	Whole‐gene deletion	—	N/A	SeLFIE	Known as PKD (PMID:23363396)	P	PVS1 + PS1	—	—	—
1 (1.0%)	c.1011C>T	p.Gly337Gly	Synonymous	Exon3	Familial	SeLFIE	Known as HM and PKD (PMID:23077024,26598494)	VUS	PM2 + BS3 + PP5	—	—	DC
1 (1.0%)	c.236C>T	p.Ser79Leu	Missense	Exon2	Familial	SeLFIE	Known as BEB (PMID:32038460)	VUS	PM2	Benign (0.015)	D (0.003)	Polymorphism
1 (1.0%)	c.640G>C	p.Ala214Pro	Missense	Exon2	Familial	SeLFIE	Known as PKD (PMID:27123484)	LB	PM2 + BS3	PrD (0.983)	D (0.022)	Polymorphism
1 (1.0%)	c.826T>A	p.Cys276Ser	Missense	Exon2	Familial	PKD	Novel	VUS	PM2 + PM5 + PM1 + PP3	PrD (0.999)	D (0.005)	DC
1 (1.0%)	c.865G>C	p.Ala289Pro	Missense	Exon2	Familial	ICCA	Novel	VUS	PM2 + PM1	PD (0.769)	D (0.012)	Polymorphism
1 (1.0%)	c.916G>A	p.Ala306Thr	Missense	Exon3	Familial	PKD	Known as SeLFIE and PKD (PMID:23063574, 30392205)	VUS	PM2 + PM5 + PM1 + PP3 + BS3	PrD (0.999)	D (0.002)	DC
1 (1.0%)	c.213_214delCA	p.Thr71Thrfs ^∗^62	Frameshift	Exon2	De novo	PKD	Novel	P	PS4 + PVS1 + PM2	—	—	DC
1 (1.0%)	c.319delG	p.Glu107Alafs ^∗^8	Frameshift	Exon2	Familial	SeLFIE	Novel	LP	PVS1 + PM2	—	—	DC
1 (1.0%)	c.514_517delTCTG	p.Ser172Leufs ^∗^3	Frameshift	Exon2	Familial	SeLFIE	Known as PKD (PMID:22101681)	P	PVS1 + PM2 + PS4	—	—	DC
1 (1.0%)	c.592_599delCCTGAGCC	p.Pro198Hisfs ^∗^24	Frameshift	Exon2	Familial	SeLFIE	Novel	LP	PVS1 + PM2	—	—	DC
1 (1.0%)	c.609_612delACCC	p.Pro204Glnfs ^∗^24	Frameshift	Exon2	Familial	PKD	Novel	LP	PVS1 + PM2	—	—	DC
1 (1.0%)	c.640delG	p.Ala214Profs ^∗^15	Frameshift	Exon2	De novo	PKD	Novel	P	PVS1 + PS2 + PM2	—	—	DC
1 (1.0%)	c.641delC	p.Ala214Alafs ^∗^15	Frameshift	Exon2	Familial	SeLFIE	Novel	P	PS4 + PS3 + PVS1	—	—	DC
1 (1.0%)	c.650delG	p.Arg217Profs ^∗^12	Frameshift	Exon2	Familial	ICCA	Known as SeLFIE, HM, and PKD (PMID:24928127, 31154286)	P	PVS1 + PM2 + PS4	—	—	DC
1 (1.0%)	c.748delC	p.Gln250Argfs ^∗^63	Frameshift	Exon2	De novo	SeLFIE	Novel	P	PVS1 + PS2 + PM2	—	—	DC
1 (1.0%)	c.363_364insT	p.Gln122Argfs ^∗^12	Frameshift	Exon2	Familial	SeLFIE	Novel	LP	PVS1 + PM2	—	—	DC
1 (1.0%)	c.516_517insT	p.Glu173Terfs ^∗^1	Frameshift	Exon2	Familial	SeLFIE	Known as ICCA and PKD (PMID:22832103)	P	PVS1 + PS3 + PM2	—	—	DC
1 (1.0%)	c.572_573insT	p.Asp191Aspfs ^∗^9	Frameshift	Exon2	Familial	SeLFIE	Novel	P	PVS1 + PS1 + PM2	—	—	DC
1 (1.0%)	c.‐65‐2A>G	—	Splice‐site	IVS1	Familial	PKD	Novel	LP	PVS1 + PM2	—	—	DC
1 (1.0%)	c.880‐34G>A	—	Splice‐site	IVS2	De novo	SeLFIE	Known as SeLFIE and PKD (PMID:26936445)	P	PS2 + PS3 + PM2	—	—	DC

*Note:* Polyphen‐2 is a tool used to predict the impact of an amino acid substitution on the structure and function of a protein. Its score ranges from 0.0 to 1.0. A score ≥ 0.909 is typically classified as “probably damaging,” a score between 0.447 and 0.909 (inclusive) as “possibly damaging,” and a score ≤ 0.446 as “benign.” SIFT predicts the deleteriousness of a missense mutation by assessing the conservation of the amino acid position in evolutionary terms. Predictions are given as scores, where a score ≤ 0.05 is classified as “deleterious” and a score > 0.05 is classified as “tolerated.” MutationTaster evaluates the disease‐causing potential of a DNA sequence variant based on features such as evolutionary conservation and splice‐site effects. It does not provide a single score but rather a direct classification, such as “disease causing,” “polymorphism,” “probably disease causing,” or “probably polymorphism.”

Abbreviations: BEB, benign essential blepharospasm; CNV, copy number variation; D, deleterious; DC, disease causing; HM, hemiplegic migraine; LB, likely benign; LP, likely pathogenic; P, pathogenic; PD, possibly damaging; PrD, probably damaging; VUS, variant of uncertain significance.

Six variants of uncertain significance were identified, including five missense variants and one synonymous variant. One of these, c.640G>C (p.Ala214Pro), was subsequently reclassified as LB based on functional evidence (Figure S1). Among the missense VUS, multisoftware predictions indicated that three (c.826T>A, c.865G>C, and c.916G>A) were potentially deleterious, whereas c.236C>T was predicted to be B. Preliminary bioinformatic analysis suggested that the three missense mutations may affect hydrogen bonding in the vicinity of the variant sites, potentially compromising structural stability (Figure S2).

The majority of P variants (76.7%) were localized to the N‐terminal cytoplasmic region of the encoded protein. Within this region, the proline‐rich domain represented a distinct mutational hotspot (Figure 2A,B) where 18 pathogenic alleles—including the most frequent frameshift insertion, c.649_650insC (p.Arg217Profs ^∗^8)—were clustered (Figure 1).

**Figure 1:**
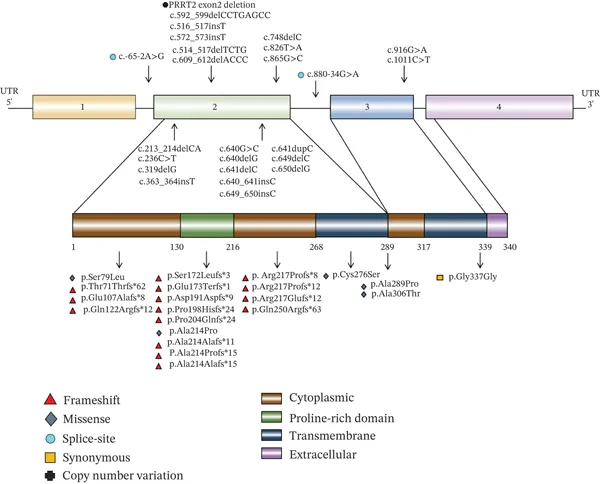
The *PRRT2* gene has 103 variant sites located as shown in the figure. The *PRRT2* gene contains four exons that encode a 340‐amino acid protein, which includes two cytoplasmic regions—the first of which contains a proline‐rich domain—two transmembrane regions, and one extracellular region. PRRT2, proline‐rich transmembrane protein 2; UTR, untranslated regions.

### 3.3. Genotype–Phenotype Correlation

LOF mutations—comprising frameshift variants, copy number variations involving *PRRT2* exon 2 deletions, whole‐gene deletions, and the canonical splice‐site variant c.‐65‐2A>G—accounted for 93.2% (96/103) of all cases and were identified in nearly every patient with SeLFIE and ICCA. All three instances of whole‐gene deletions occurred exclusively in the SeLFIE cohort. In the isolated PKD group, LOF mutations remained prevalent, affecting 90.9% (20/22) of patients.

The proportion of structural brain abnormalities declined progressively across the SeLFIE, ICCA, and PKD groups. Most EEG and MRI abnormalities were associated with mutations located in the N‐terminal cytoplasmic region and the proline‐rich domain of the encoded protein (Figure 2C). Notably, all EEG recordings in the PKD group were normal. Additionally, every child in our cohort presenting with intellectual disability or developmental delay carried exclusively LOF mutations.

**Figure 2:**
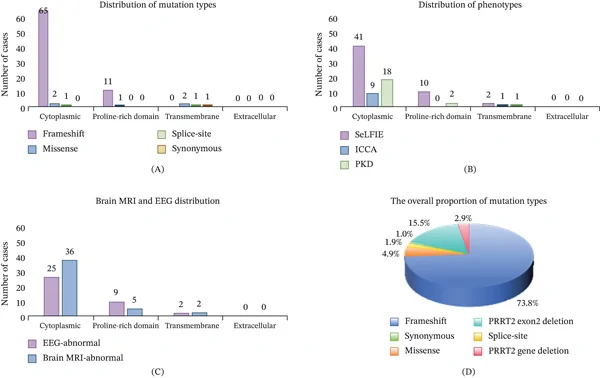
The association between *PRRT2* mutation spectrum and clinical manifestations was analyzed. (A) The distribution of the number of mutations in different protein domains (cytoplasmic region, proline‐rich domain, transmembrane region, and extracellular region) is shown. (B) Distribution of clinical phenotypes (SeLFIE, ICCA, and PKD) corresponding to different protein domain mutations. (C) Comparison of the number of brain MRI abnormalities and EEG abnormalities in patients with different protein domain mutations. (D) The percentage of different types of gene mutations (frameshift, missense, splice site, synonymous mutation, and *PRRT2* exon and overall gene deletion).

With regard to treatment outcomes, responses followed a genotypic–phenotypic gradient: Seizure freedom was achieved in 94.3% of SeLFIE patients, compared with only 72.7% in the ICCA group. This observation suggests that the presence of a dyskinesia phenotype, even when associated with the same LOF mutation, may render pharmacological control more challenging. In contrast, PKD responded favorably to antidyskinesia therapy, with an effectiveness rate of 95.5%.

## 4. Discussion

The *PRRT2*‐related disease spectrum exhibits high genetic heterogeneity. Through a comprehensive analysis of 103 Chinese pediatric patients harboring *PRRT2* mutations, this study systematically delineates the clinical and genetic landscape of this disease spectrum. Our findings reinforce the prevailing view that *PRRT2*‐related disorders are primarily driven by haploinsufficiency [10, 11]. More importantly, by constructing a continuous gradient model that links genotype to clinical phenotype and, subsequently, to treatment response, we offer novel insights into the underlying pathophysiology and provide a framework for advancing personalized therapeutic strategies.

In our cohort, LOF variants constituted a substantial majority of mutations (93.2%), a figure that aligns closely with findings reported by several other research groups [12–14]. Of particular note, 76.7% of all mutations—including the most frequently observed alteration, c.649_650insC (p.Arg217Profs ^∗^8)—were densely concentrated within the proline‐rich domain (PRD) located in the N‐terminal cytoplasmic region of the PRRT2 encoded protein. The PRD is known to function as a protein–protein interaction scaffold, facilitating associations with presynaptic multiprotein complexes [15, 16]. Previous work has established that PRRT2 regulates synaptic vesicle exocytosis and neuronal excitability through its binding to SNAP25 and synaptotagmin 1/2 [2]. Therefore, disruption of the PRD is likely to compromise the correct membrane anchoring and functional integrity of PRRT2 at the presynaptic active zone, thereby disturbing the equilibrium between excitatory and inhibitory neurotransmitter release. This provides a structural biology explanation for the high pathogenicity of LOF mutations. Collectively, our data identify the PRD as a definitive mutational hotspot, reinforcing its critical role in preserving normal PRRT2 physiological function.

This study reveals that *PRRT2*‐associated phenotypes are not discrete entities but rather form a continuous spectrum influenced by the severity of the genotype. At one end of this spectrum lies SeLFIE, which is almost exclusively composed of LOF mutations, including all three cases with full gene deletions in our cohort. This indicates that complete or near‐complete loss of PRRT2 protein function is a prerequisite for the infant brain′s susceptibility to convulsive seizures. The immature brain networks in infancy, with particularly vulnerable inhibitory circuits, may be especially sensitive to such functional loss [17, 18]. The ICCA phenotype is also predominantly caused by LOF mutations, yet its unique biphasic course suggests more complex mechanisms. One plausible explanation involves age‐dependent neural network compensation and plasticity [19, 20]. During infancy, severe *PRRT2* deficiency is sufficient to lower the seizure threshold, leading to epilepsy. As brain networks mature and compensatory mechanisms develop, seizures may resolve; however, latent deficits in fine motor regulation within the basal ganglia‐thalamocortical circuitry persist, becoming unmasked in childhood by specific triggers such as sudden movement, manifesting as paroxysmal dyskinesia [21, 22].

In our cohort, LOF mutations remained the predominant class across all clinical phenotypes (SeLFIE: 94.3%; ICCA: 90.9%; PKD: 90.9%), with no statistically significant difference in the distribution of LOF versus non‐LOF variants among the three groups (*p* = 0.820). Missense mutations may produce proteins that retain partial correct folding and residual interaction capacity. Previous studies have suggested that residual activity from missense variants may be sufficient to protect the developing brain from severe convulsive seizures during early life yet inadequate to maintain the stability of motor circuits when subjected to high‐frequency activation in later childhood [23, 24]. Our bioinformatic predictions, supported by protein structure modeling, suggest that these missense mutations may disrupt local hydrogen‐bonding networks, thereby compromising overall structural stability [25, 26]. Furthermore, previous studies have also indicated that phenotypes associated with non‐LOF mutations are more heterogeneous, and their clinical consequences may be highly dependent on the specific genomic context [27].

Functional assays demonstrated that the missense variant c.640G>C (p.Ala214Pro) retains normal protein expression and plasma membrane localization [9], consistent with its reclassification as LB. In our cohort, the individual harboring this variant presented with uncomplicated SeLFIE and achieved good seizure control, which is consistent with the overall phenotypic spectrum observed in patients with non‐LOF variants. The synonymous variant c.1011C>T was shown to have no effect on splicing, supporting its retention as a VUS; this patient also presented with uncomplicated SeLFIE [8]. In contrast, the splice‐site variant c.880‐34G>A was confirmed to disrupt normal splicing through activation of a cryptic intronic acceptor site, resulting in a frameshift and premature termination codon [8]. The patient harboring this variant presented with SeLFIE, consistent with the observation that LOF variants account for the vast majority of SeLFIE cases in our entire cohort.

Our clinical data further support the genotype–phenotype gradient. The rates of abnormal EEG and brain MRI findings progressively decreased from SeLFIE to ICCA and to PKD. All PKD patients exhibited normal EEG, with the lowest rate of MRI abnormalities. Regarding treatment response, SeLFIE demonstrated excellent responsiveness to ASMs, whereas seizure control in ICCA proved more challenging, suggesting broader or more complex underlying neural network pathology [28, 29]. In contrast, PKD responded remarkably well to low‐dose sodium channel blockers such as oxcarbazepine, likely because the medication further stabilizes the relatively intact neural circuits maintained by residual PRRT2 function [30, 31].

The gradient framework proposed in this study offers a valuable basis for refining genetic counseling and clinical management approaches. For families harboring typical LOF variants—especially those situated within the PRD region—it is important to convey that affected offspring face a substantial likelihood of manifesting epilepsy‐related phenotypes, with SeLFIE being the most prominent presentation and ICCA representing a possible additional outcome. For families with non‐LOF mutations, it can be anticipated that the phenotype may be limited to PKD or present with relatively mild symptoms and the prognosis is generally very favorable. This can provide more positive psychological support for these families. For all children with epilepsy at onset, regardless of their initial genotype, families should be informed about the possibility of phenotypic conversion, and long‐term neurodevelopmental follow‐up is recommended.

## 5. Limitations

The limitations of this study include its single‐center retrospective design, which may introduce referral bias. The sample sizes for PKD and ICCA within the cohort were relatively small, potentially limiting the statistical power to detect certain significant differences. Future multicenter, prospective long‐term studies, combined with functional experiments, are warranted to further validate the pathogenic mechanisms of the newly identified mutations.

## 6. Conclusion

In summary, this study establishes, through a multidimensional analysis of 103 patients, a clear genotype–phenotype gradient model based on PRRT2 protein residual activity within the *PRRT2*‐associated disease spectrum. This model links the severity of LOF mutations to the clinical spectrum ranging from SeLFIE and ICCA to PKD, supported by findings from auxiliary examinations and treatment responses. This framework not only deepens our understanding of the molecular mechanisms of the disease but, more importantly, provides clinicians with a crucial practical guide for precise diagnosis, prognosis assessment, and personalized genetic counseling for families.

## Author Contributions

Y.L., G.Z., and A.Z. contributed to the conception and design of the study. B.W., B.Z., X.L., and G.Y. contributed to the acquisition and analysis of data. S.W., X.M., G.Y., and M.H. contributed to drafting the manuscript and preparing the figures. S.W., X.M., and G.Y. have contributed to the work equally and should be regarded as cofirst authors. Y.L., A.Z., and G.Z. jointly supervised this work.

## Funding

This study is funded by the Natural Science Foundation of Jiangsu Province (doi:10.13039/501100004608, BK20241732) and the Science and Technology Innovation Support Program for High‐Level Hospitals in Children′s Hospital of Nanjing Medical University (KJCXZ2024004).

## Ethics Statement

This is an observational study. The Research Ethics Committee of Children′s Hospital Affiliated to Nanjing Medical University has confirmed that no ethical approval is required.

## Consent

Written informed consent was obtained from the parents.

## Conflicts of Interest

The authors declare no conflicts of interest.

## Supporting Information

Additional supporting information can be found online in the Supporting Information section.

## Supporting information


**Supporting Information 1** Figure S1 Pedigree analysis of six variants initially classified as variants of uncertain significance (five missense mutations and one synonymous mutation). c.640G>C (p.Ala214Pro) was subsequently reclassified as likely benign based on functional evidence.

**Supporting Information 2** Figure S2 Conservation analysis and structural modeling of five *PRRT2* missense mutations. Analysis of sequence conservation across different species for five missense mutation sites in the PRRT2 protein, and structural modeling of the PRRT2 homologous protein using PyMOL software, with prediction of changes in the secondary and tertiary structure before and after mutation.

## Data Availability

The data that support the findings of this study are available from the corresponding author upon reasonable request.
